# Supplementary material for: Co-occurrence of diabetes and depression in the U.S
Source: PLoS One. 2020 Jun 25;15(6):e0234718. doi: 10.1371/journal.pone.0234718 (PMC7316423; doi:10.1371/journal.pone.0234718)
Supplement: Attachment — Submitted filename: Response to reviewers.docx [file pone.0234718.s001.docx]

**Editor’s comments**

1. Please ensure that your manuscript meets PLOS ONE's style requirements, including those for file naming. The PLOS ONE style templates can be found at

<http://www.journals.plos.org/plosone/s/file?id=wjVg/PLOSOne_formatting_sample_main_body.pdf> and <http://www.journals.plos.org/plosone/s/file?id=ba62/PLOSOne_formatting_sample_title_authors_affiliations.pdf>

**Answer.** Thank you. The title page now follows PLOS ONE style template. My affiliation has also been updated. In the main text – all headings follow the names used in the guideline, i.e., I now call “introduction” what was previously defined as “background”.

2. We noticed you have some minor occurrence of overlapping text with the following previous publication(s), which needs to be addressed:

Bogner, Hillary R., and Heather F. de Vries McClintock. "Costs of coexisting depression and diabetes." (2016): 594-595. In your revision ensure you cite all your sources (including your own works), and quote or rephrase any duplicated text outside the methods section. Further consideration is dependent on these concerns being addressed.

**Answer.** Any overlapping text is accidental. I used here the software that I use to evaluate plagiarism in my students’ essays. The only parallel I found is regarding this section: “The global burden of disease study … prevalence” I have deleted that sentence because it does not change in any meaningful way the arguments in the text and I have added instead other evidence from the literature to highlight the importance of the paper.

If you have specific text suggestions that have not been captured by software, please kindly point those to me as I am unable to identify them.

3. We note that you have stated that you will provide repository information for your data at acceptance. Should your manuscript be accepted for publication, we will hold it until you provide the relevant accession numbers or DOIs necessary to access your data. If you wish to make changes to your Data Availability statement, please describe these changes in your cover letter and we will update your Data Availability statement to reflect the information you provide.

**Answer.** The dataset I have used in my analysis is publicly available. Reference 16 provides the links to access the data.

4. Thank you for stating the following in the Financial Disclosure section:"The author received no specific funding for this work."

Thank you for stating the following in the Competing Interests section:"The author declares that no competing interests exist."

We note that one or more of the authors are employed by a commercial company: "IMPAQ International,"

**Answer.** My former employer, IMPAQ international is not a commercial company but a (for profit) research organization. I was a senior economist there, and all researchers of that institution as well as similar ones have to undergo background checks that guarantee they do not have conflicts of interest. More information about my former employer is available here: <https://www.impaqint.com/>. While the paper was under review I have become a professor at Georgetown University.

a)Please provide an amended Funding Statement declaring this commercial affiliation, as well as a statement regarding the Role of Funders in your study. If the funding organization did not play a role in the study design, data collection and analysis, decision to publish, or preparation of the manuscript and only provided financial support in the form of authors' salaries and/or research materials, please review your statements relating to the author contributions, and ensure you have specifically and accurately indicated the role(s) that these authors had in your study. You can update author roles in the Author Contributions section of the online submission form.

**Answer.** All parts of the study (the study design, data collection and analysis, decision to publish, or preparation of the manuscript) were done on my own time and not during IMPAQ’s working hours. Therefore, IMPAQ was not the “funding organization” as my salary then reflected the hours spent on contract work and did not cover projects done in my own time. Also please note my change in affiliation to a university institution.

Please also include the following statement within your amended Funding Statement.

“The funder provided support in the form of salaries for authors [insert relevant initials], but did not have any additional role in the study design, data collection and analysis, decision to publish, or preparation of the manuscript. The specific roles of these authors are articulated in the ‘author contributions’ section.”

**Answer.** Both at IMPAQ (previously) and at Georgetown University (not) I (dis not) do not get a salary to write papers. I write papers on my own time and interest-- I have no conflict of interests with any organization that might draw any benefit from my independent research. I am happy to state the truth and to explain further my circumstances, with the amount of detail the editors suggest would be appropriate. However, the suggested statement does not reflect my situation.

If your commercial affiliation did play a role in your study, please state and explain this role within your updated Funding Statement.

**Answer.** As aforementioned, my former employer is not a commercial company but an interdependent research organization whereby employees have to be able to demonstrate no COIs. My current employer is a University, with the same requirements.

b). Please also provide an updated Competing Interests Statement declaring this commercial affiliation along with any other relevant declarations relating to employment, consultancy, patents, products in development, or marketed products, etc.

**Answer.** I do not have now (or ever had) a commercial affiliation. I hope my previous answers clarify any confusion related to my former employer.

Within your Competing Interests Statement, please confirm that this commercial affiliation does not alter your adherence to all PLOS ONE policies on sharing data and materials by including the following statement: "This does not alter our adherence to PLOS ONE policies on sharing data and materials.” (as detailed online in our guide for authors http://journals.plos.org/plosone/s/competing-interests) . If this adherence statement is not accurate and there are restrictions on sharing of data and/or materials, please state these. Please note that we cannot proceed with consideration of your article until this information has been declared.

**Answer.** The data I used (BRFSS) in my analysis is publicly available. I have included a link in the paper (reference number 16) to where readers can download the data.

Please include both an updated Funding Statement and Competing Interests Statement in your cover letter. We will change the online submission form on your behalf.

Additional Editor Comments:

Title: I suggest modifying title. The “risk” factors described are weakly indicative of “co-occurrence” and recommendations provided are not robust enough to include 'risk' within the title are not robust enough to include 'risk' within the title.

**Answer.** As requested, I deleted from the title “How can we target those at risk better?”

Abstract: Please provide full name for BRFSS

**Answer.** Done. The text in the abstract now reads “Behavioral Risk Factor Surveillance System”

Background: Good background overall. Can make more a bit more detail in the background on existing studies of state level variation in the distribution of the conditions of interest and what the current report may add.

**Answer.** Thank you. More details in the background has been added on studies that state national level variation. There are no peer reviewed studies looking at differences across states.

Design: The description in this section is one of statistical analytic approach rather than design. This needs rewriting.

**Answer.** Following the editor’s advice, I now use the term “statistical approach” rather than design.

Results: Correlation of 0.17 is very low despite statistical significance. This needs to be reflected upon. All “exhibit” should be relabelled as Figure. Table: Would be helpful for readers to be explicit about what the coefficients are representing.

**Answer.** Thank you. All exhibits have now been re-labeled as figures and tables.

In the text description of “Exhibit 3” (page 7, line 21-22), the detailed reference to the axis should go as an explanatory note under the exhibit itself.

**Answer.** Thank you. A note has now been added under Figure 1, as requested.

Discussion: Does not pay attention in any depth to the state level differences and why comorbidity at the state level may be explained. It would have also been good to have concluding remarks or reflections at the end.

**Answer.** Thank you. In the results and discussion, differences across states are explained in depth – however, understanding why these differences come about are out of the scope of this paper, as the data available does not allow us to answer that question.

Reviewers' comments:

Reviewer's Responses to Questions

Comments to the Author

1. Is the manuscript technically sound, and do the data support the conclusions?

The manuscript must describe a technically sound piece of scientific research with data that supports the conclusions. Experiments must have been conducted rigorously, with appropriate controls, replication, and sample sizes. The conclusions must be drawn appropriately based on the data presented.

Reviewer #1: Yes

Reviewer #2: Partly

2. Has the statistical analysis been performed appropriately and rigorously?

Reviewer #1: I Don't Know

Reviewer #2: Yes

3. Have the authors made all data underlying the findings in their manuscript fully available?

The PLOS Data policy requires authors to make all data underlying the findings described in their manuscript fully available without restriction, with rare exception (please refer to the Data Availability Statement in the manuscript PDF file). The data should be provided as part of the manuscript or its supporting information, or deposited to a public repository. For example, in addition to summary statistics, the data points behind means, medians and variance measures should be available. If there are restrictions on publicly sharing data—e.g. participant privacy or use of data from a third party—those must be specified.

Reviewer #1: Yes

Reviewer #2: Yes

4. Is the manuscript presented in an intelligible fashion and written in standard English?

PLOS ONE does not copyedit accepted manuscripts, so the language in submitted articles must be clear, correct, and unambiguous. Any typographical or grammatical errors should be corrected at revision, so please note any specific errors here.

Reviewer #1: Yes

Reviewer #2: Yes

5. Review Comments to the Author

Please use the space provided to explain your answers to the questions above. You may also include additional comments for the author, including concerns about dual publication, research ethics, or publication ethics. (Please upload your review as an attachment if it exceeds 20,000 characters)

Reviewer #1: I would like more information about why the authors used the data analysis they chose. I also feel that the one scatter plot on individuals could be re-done . it is impossible to decipher. I also feel the conclusion is weak and could be re-done to highlight why the article is important

**Answer.** Reviewer #1 does not provide any guidance on how s/he would like Figure 2 to be changed or why it is hard for s/he to understand it. I added footnoted to assist the reader with interpretation.

Reviewer #2: The manuscript entitled 'Co-occurrence of diabetes and depression in the U.S.: How can we target those at risk better?' with the aim to evaluate demographic and socio-economic indicators associated with both depression and diabetes at the macro (across-states) and micro level (individuals)'

This is quite an interesting study. However, the manuscript requires improvement based on the following comments.

Abstracts Page 2 Line 12, BMI >30 or BMI BMI≥3030? Likewise in discussion section i.e. Page 8 Line 17, 19, Page 9 Line 1.

**Answer.** Thank you. Obesity was denoted with BMI≥30. This has been amended in the text and made consistent with the computations.

Try to avoid using active sentence i.e 'I' (Page 5 Line 7,22; Page 6 Line 10)

**Answer.** All active sentences were avoided. On page 5, I now use “This analysis uses”. On page 6, line 2, I changed the previously active voice into passive voice: “ Women….were excluded from the sample”. On page 6, line 13, I now write “All analyses control for…”

Page 5, more information/description to be provided on BRFSS such as data type, type of measurement/ test/ inventories/ questionnaires etc

i.e measure for diabetes, depression etc

Page 5 Line 10-13, more information to be provided.

**Answer.** I added clarification on the type of data available in BRFSS as requested

Subjects Page 5 Line 16-23, this section requires revision to state that these subjects (depression and/or diabetes) were identified in BRFSS via the two questions which were found in BRFSS.

**Answer.** The fact that the questions are found in BRFSS is now stated in the text as requested

Page 5 Line 16-17, to clearly state how many subjects were analyzed and to state if any missing data occurred in the database that was used in the analysis.

**Answer.** The information requested is stated on page 5, line 21. 477,665 respondents answered the question about diabetes and depression. 40,921.00 (or 9% of the sample) did not have complete information on all the demographic characteristics the study included and therefore were excluded from the sample. I re-edited the section to make the exposition clearer.

Design Page 6 Line 6, 7, the words 'could be', 'might be' to be avoided.

**Answer.** “Could be” and “might be” gave been substituted with “would”

Page 6 Line 13, 14, 16, 17, the equation to be labelled.

**Answer.** The equations (previously in line 13 and 14) were label as the reviewer suggested. The correlation matrix was not labelled because that is NOT an equation not it representants two different arguments like the reviewer seems to indicate. It is one single argument.

More information to be provided on the statistical analysis such as type of regression analysis, model fit, level of acceptance significance, 1 or 2 tailed test etc and description on how the analyses were performed on state and individual level. The statistical software STATA including the publisher name and version to be stated.

**Answer.** On page 5, it was already stated that the statistical software used is STATA 15. It is also stated that I use a bivariate normal model. Standard errors, P-values and confidence intervals are presented in Tables 1 and 2. The study is descriptive and does not perform differences across cohorts therefore the comment being made on tests is not relevant in this case.

Results Page 7 Line 2, 17% to be replaced with 17.2%.

**Answer.** The change requested was done**.**

The word Exhibit to be replaced with word Table.

**Answer.** The change requested was done**.**

Exhibit 1, technically p value cannot be written as 0.000 (to use symbol < to denote less). Denote what 'omitted' and 'ρ' refers to in the table footnote. CIL and CIU to be written as 95% CI (Lower, Upper). P>[t] to be written as P. Symbol >= to be replaced with symbol ≥. The category for BMI and veteran to be stated in the table footnote. Likewise with Exhibit 2. dy/dx to be denoted in table footnote.

**Answer.** 0.000 was replaced through with <0.0001 as requested. A footnote has been added under table 1, as requested. 95% CI (Lower, Upper) has been written in lieu of CIL CIU. The Symbol >= has been replace.

A footnote for dy/dx has been added in the footnote under Exhibit 2 – now labelled as Table 2. Note however that the table already explains that dy/dx means: “marginal probability”.

Page 7 Line 22 - Page 8 Line 11, total states added up to 51. On Page 5 Line 16, it was stated 50.

**Answer.** It is 50 states plus DC. The counts add up to 51 and are consistent with the description.

Page 9 Line 10 and 12, CI to be written as 95%CI.

**Answer.** The change requested was added.

Overall, the writeup requires revision in terms of flow, clarity and more information to be provided.

6. PLOS authors have the option to publish the peer review history of their article (what does this mean?). If published, this will include your full peer review and any attached files.

If you choose “no”, your identity will remain anonymous but your review may still be made public.

Both referees had chosen not to be identified.
