# Supplementary material for: Co-occurrence of diabetes and depression in the U.S
Source: PLoS One. 2020 Jun 25;15(6):e0234718. doi: 10.1371/journal.pone.0234718 (PMC7316423; doi:10.1371/journal.pone.0234718)
Supplement: Attachment — Submitted filename: response to referees.docx [file pone.0234718.s002.docx]

PONE-D-19-22449R1
Co-occurrence of diabetes and depression in the U.S.
PLOS ONE

- Table 1, for Did not graduate from high school, the figures 33.9% 41.7% , 16.8% 23.9% to be aligned parallel with other figures.

**Answer:** Thank you. The table was re-formatted

- Table 2, Diabetes only P(y1=1, y2=0) (Non hispanic black, hispanic, native, others) typo <.0001<.0001.

**Answer:** Thank you. The typo has now been corrected.

- The word marginal probability in the first column to be replaced with variables.

**Answer:** Thank you. This was changed.

- STATA which was stated for 'The survey design was specified as follows, using Stata 15: svyset [pweight=_LLCPWT], strata(_STSTR) psu(_PSU) to be stated in statistical analysis section with citation 'STATA: Release 15. College Station, TX: StataCorp LLC' and the level of accepted statistical significant.

**Answer:** The STATA citation has been added and all the references have been reformatted.

- Some references did not conform to the journal format.

**Answer:** All references have now been formatted using the ICMJE style
